# Prevalence and associated factors of myopia among rural school students in Chia-Yi, Taiwan

**DOI:** 10.1186/s12886-020-01590-y

**Published:** 2020-08-05

**Authors:** Li-Ju Lai, Wei-Hsiu Hsu, Tao-Hsin Tung

**Affiliations:** 1 Ophthalmology, Universal Eye centre, Chia-Yi, Taiwan; 2 School of Medicine, Chang Gung University, Lin-ko, Taoyuan City, Taiwan; 3 Department of Orthopedics Surgery, Chang Gung Memorial Hospital, No. 6, West section, Chia-Pu Road, Pu-Zih City, Chia-Yi County Taiwan 61363; 4 Department of Medical Research and Education, Cheng-Hsin General Hospital, Taipei, Taiwan

**Keywords:** Myopia, School students, Rural, Taiwan, Heterophoria

## Abstract

**Background:**

The prevalence of myopia has increased rapidly worldwide over the past few decades. The aim of this study was to evaluate the prevalence and associated risk factors for myopia in elementary and junior high school students in Chia-Yi, Taiwan.

**Methods:**

We included 5417 students in total from Grade 1–6 (*n* = 4763) and Grade 7–9 (*n* = 654) from Chia Yi County in this population-based study. The students underwent noncycloplegic autorefractometry and an interview with a structured questionnaire.

**Results:**

For this study population, the prevalence of myopia and high myopia was 42.0 and 2.0%, respectively, revealing a statistically significant increase with increasing age (*p* < 0.05). Junior high school students (aged 13–15) showed a greater prevalence of myopia than elementary school students (aged 7–12) (55.8% vs. 40.1%, respectively, *p* < 0.001). Multiple logistic regression analysis showed that associated factors of myopia were body height (odds ratio [OR]: 1.05, 95% confidence interval [CI]: 1.05–1.06), body mass index (OR: 0.98, 95% CI: 0.96–1.00), and ocular alignment (horizontal heterophoria vs. orthophoria, OR: 2.37, 95% CI: 2.08–2.70; tropia vs. orthophoria, OR: 1.94, 95% CI: 1.50–2.52) for elementary school students, whereas in junior high school students, they included body height (OR: 1.02, 95% CI: 1.01–1.04) and ocular alignment (heterophoria vs. orthophoria, OR: 2.20, 95% CI: 1.56–3.10).

**Conclusions:**

This study provided epidemiological data on myopia in rural school students in Chia-Yi, Taiwan, and demonstrated the association between heterophoria and myopia. Correction of refractive errors in the students remained a challenge.

## Background

The prevalence of myopia has increased rapidly worldwide over the past few decades, particularly in East Asia [1–4]. Moreover, 10–20% of such patients progress to high myopia, with an increased risk of complications such as retinal detachment, glaucoma, cataract, and pathological myopia [5, 6]. The prevalence of myopia and high myopia has been estimated to increase significantly from 2000 to 2050 [7]. Recently, epidemiological studies performed to investigate the prevalence and risk factors for myopia have provided information on modifiable risk factors for myopia, including duration of outdoor activities, duration of near work, and vision screening [8–12]. Meanwhile, routine screening can detect this disorder early to prompt appropriate clinical intervention.

In Taiwan, the prevalence rates of myopia in elementary school children aged 7 years were 5.8, 3.0, 6.6, 12.0, 20.0, 19.6, and 17.9% in 1983, 1986 1990, 1995, 2000, 2006, and 2010, respectively. The rates for the same years among children aged 12 years were correspondingly 36.7, 27.5, 35.2, 55.5, 61.0, 62, and 66%. At the junior high school level, the rates for the corresponding years were 64.2, 61.6, 74.0, 76.0, and 81.0% [1]. The Ministry of Education in Taiwan launched a myopia prevention programme for young children in 2009. This intervention covers preventive measures, such as outdoor activities for 2 h every day, classroom clearance between classes for outdoor activities, 10 min of disruption in near vision every 30 min, vision screening by a school nurse twice a year, and cycloplegic refractometry to screen high- risk groups for myopia. However, exposure to smartphone and smart devices among children can lead to long hours of near work. Thus, myopia prevention is a challenge [8].

We therefore performed this population-based study to understand the prevalence of myopia and risk factors for myopia, including horizontal heterophoria, intraocular pressure, and sleep duration, among school children in the rural setting of Chia-Yi, a prefecture, in south Taiwan.

## Methods

### Participants

We recruited students of 33 elementary schools and 3 junior high schools from July 2014 to June 2015 in Chia-Yi, Taiwan, for this cross-sectional study. In total, 5417 participants underwent surveys of refraction and eye health as well as associated life style investigations. We included two cohorts, namely the age groups of 7–12 years and 13–15 years, that comprised students from elementary and junior high schools, respectively. We contacted the local administration of the Education and School Board to request their cooperation. The Institutional Review Board of Chang Gung Foundation (102-4827B) approved our study, and we followed the tenets of the Declaration of Helsinki. Written consent was obtained from the parents or guardians of all children before the study.

### Eye examination

We used an examination environment setting of a classroom with blackout curtains for all the participants. The same investigating team administered all survey questionnaires and examinations to reduce individual errors. The body weight and height were measured. Gender, sleep duration and eye related symptoms was obtained through questionnaire. Noncycloplegic refractive errors were assessed using an auto-refractor (Autorefractometer, ARK-1, Nidek Co.,LTD., Aichi, Japan) [9]. Children presenting with a history of ocular and physical pathology, strabismus, and amblyopia were excluded. We obtained this medical data through questionnaire from children’s parents. (Supplementary doc. 1) The definition of refractive errors was based on spherical equivalent (SE) refraction calculated as the spherical dioptre plus one half of the cylindrical dioptre. An SE of ≤ − 1.0 D in one or both eyes was defined as myopia and categorised into low myopia (from − 1.0 D to − 6.0 D) and high myopia (≤ − 6.0 D). We evaluated ocular alignment using corneal reflections observations (Hirschberg test), followed by the monocular cover–uncover test. Briefly, one eye of the participant was covered, and the examiner looked for any movement in the opposite uncovered eye. Tropia was indicated by such movement. While heterophoria was indicated in case of movement of the covered eye when the cover was applied and movement in the opposite direction (a fusional movement) as the cover was removed. If the patient had heterophoria, the eye would be straight before and after the cover–uncover test. The deviation appeared during the test because of the interruption of binocular vision. We included both exophoria and esophoria in the horizontal heterophoria group for further analysis. We analysed the risk factors and prevalence of myopia and high myopia for both children with and without myopia and presented the adjusted odds ratio values for all risk factors.

### Statistical analysis

Statistical analysis was conducted using Statistical Package for the Social Sciences for Windows (v 20.0; SPSS Inc., Chicago, IL, USA); data are presented as numbers (%) and mean ± SD for fractions and continuous variables, respectively, as appropriate, and myopia data are presented as prevalence (95% confidence interval [CI]). The Pearson correlation test measured the relation between age and prevalence as well as SE refractive error. Univariate logistic analysis regression was adopted to assess differences in categorical variables. Multinomial logistic regression is the extension of the (binary) logistic regression in which the categorical dependent outcome has more than two levels. This method was also performed to provide a set of coefficients for each of the two comparisons of myopia and to investigate the independence of factors associated with the prevalence of myopia. Significance was set at *p* < 0.05.

## Results

We recruited 5417 participants in total aged 7–15 years in the present study; 4763 and 654 participants were included in the elementary and junior high school groups, respectively (Table 1). Myopia was prevalent in 40.1 and 55.8% of elementary and junior high school groups, respectively (*p* < 0.001) Regarding ocular alignment, 40.4 and 42.0% of the elementary and junior high school groups showed horizontal heterophoria, respectively (*p* = 0.68). The junior high school group showed more SE and higher incidence of myopia than the elementary school group (*p* < 0.001) (Table 1). The distribution of ocular alignment was interestingly similar in both groups.

**Table 1:** Comparison of baseline characteristics between students of elementary and junior high schools (*n* = 5417)

	Elementary school (aged 7–12) (*n* = 4763)	Junior high school (aged 13–15) (*n* = 654)	*P*-value
	mean ± SD or n (%)	mean ± SD or n (%)	
Male	2491 (52.3)	368 (56.3)	0.03
Body Height (cm)	136 ± 12	157 ± 11	< 0.001
BMI (kg/m^2^)	18.8 ± 4.1	21.3 ± 4.7	< 0.001
Spherical Equivalent (SE), OD (diopter)	− 1.06 ± 1.72	−1.86 ± 1.99	< 0.001
Intraocular pressure (IOP),OD (mmHg)	17.2 ± 4.3	17.27 ± 4.4	0.66
Sleep duration, ≧8 h/day	4045 (84.9)	418 (63.9)	< 0.001
Ocular alignment			0.68
Orthophoria	2518 (52.9)	338 (51.7)	
Horizontal heterophoria	1923 (40.4)	275 (42.0)	
Tropia	332 (6.8)	41 (6.3)	
Myopia			< 0.001
Hyperopia	51 (1.1)	0 (0.0)	
No Myopia	2721 (57.1)	262 (40.1)	
Myopia	1910 (40.1)	365 (55.8)	
High Myopia	81 (1.7)	27 (4.1)	

**Fig. 1:**
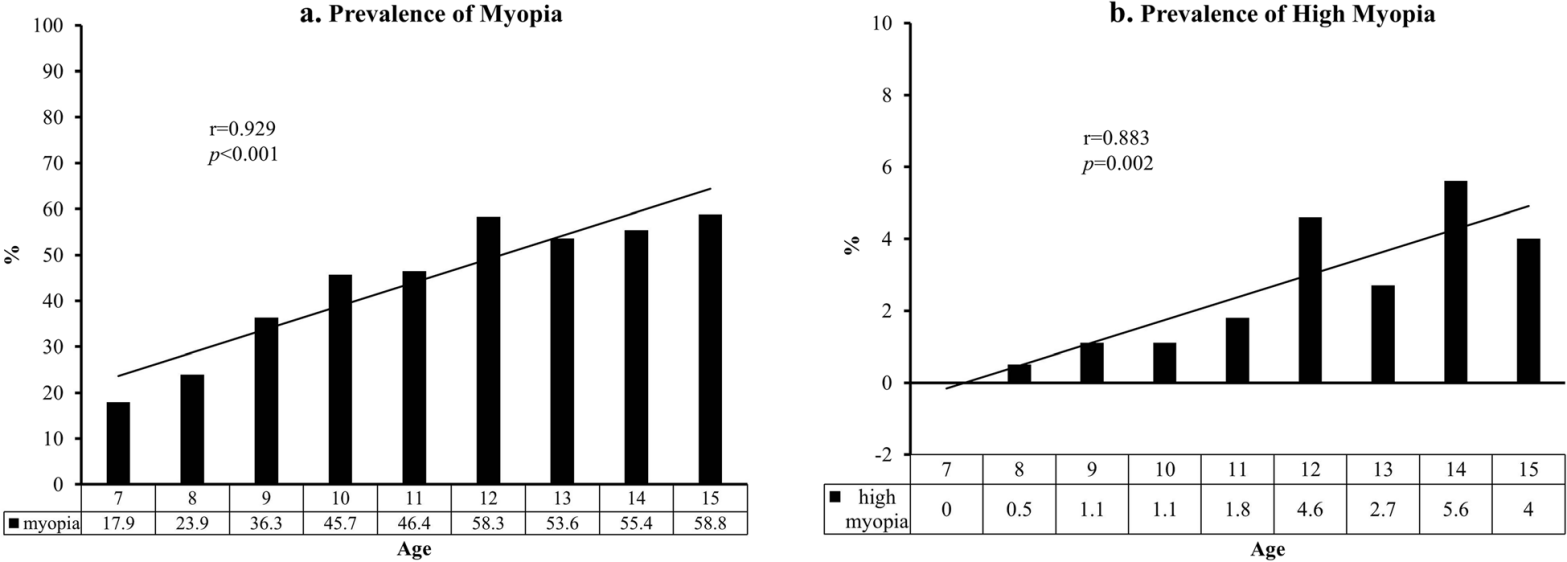
**a**. Prevalence of Myopia. **b**. Prevalence of High Myopia

**Fig. 2:**
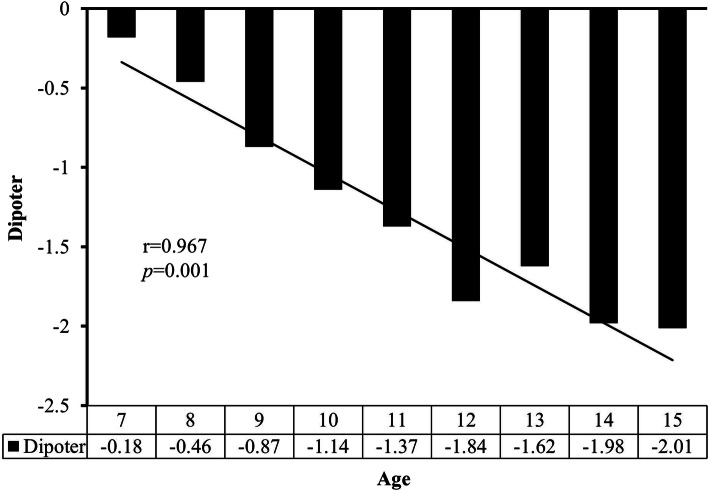
Mean of spherical equivalent refractive error stratified by age

The prevalence rates of myopia and high myopia increased with age (p < 0.001) (Fig. 1a and b), along with the SE refractive error (*p* < 0.001) (Fig. 2). Univariate analyses indicated body height, body mass index, intraocular pressure, and ocular alignment as myopia-associated factors in the elementary school group (Table 2). Similar factors were also found for high myopia, and gender was an additional factor. In the junior high school group, body height, body mass index, and ocular alignment significantly affected the development of myopia, whereas only body mass index and ocular alignment were the associated factors of high myopia.

**Table 2:** Univariate analysis for comparison of characteristics in myopia and high myopia groups (*n* = 5417)

	Myopia (*n* = 1910) vs. No Myopia (*n* = 2721)		High Myopia (*n* = 81) vs. No Myopia (*n* = 2721)	
	OR	95%CI	OR	95%CI
**Elementary school (aged 7–12) (*n*** **= 4763)**				
Gender (Female vs. Male)	1.05	0.93–1.18	1.83	1.16–2.88
Body Height (cm)	1.05	1.04–1.05	1.09	1.07–1.12
BMI (kg/m^2^)	1.05	1.04–1.07	1.10	1.05–1.16
Intraocular pressure (IOP),OD (mmHg)	1.03	1.01–1.04	1.05	1.00–1.11
Sleep(≧8 h/day vs. < 8 h/day)	1.01	0.86–1.19	0.58	0.34–0.97
Headache (yes vs. no)	1.16	0.93–1.45	0.52	0.16–1.68
Horizontal heterophoria vs. Orthophoria	2.28	2.02–2.58	9.36	4.87–17.9
Tropia vs. Orthophoria	1.84	1.44–2.35	15.7	7.20–34.6
	Myopia (*n* = 365) vs. No Myopia (*n* = 262)		High Myopia (*n* = 27) vs. No Myopia (*n* = 262)	
	OR	95%CI	OR	95%CI
**Junior high school (aged 13–15) (*n*** **= 654)**				
Gender (Female vs. Male)	0.91	0.66–1.25	0.71	0.31–1.60
Body Height (cm)	1.02	1.01–1.04	1.03	0.99–1.08
BMI (kg/m^2^)	1.05	1.01–1.09	1.10	1.02–1.19
Intraocular pressure (IOP),OD (mmHg)	1.01	0.97–1.04	0.98	0.89–1.07
Sleep(≧8 h. vs. < 8 h.)	1.23	0.89–1.71	1.85	0.76–4.53
Headache (yes vs. no)	1.65	0.71–3.85	1.22	2.35–18.8
Horizontal heterophoria vs. Orthophoria	2.24	1.59–3.14	6.64	2.35–18.75
Tropia vs. Orthophoria	1.72	0.84–3.54	15.5	4.17–57.7

The effect of independent associated risk factors on myopia and high myopia was investigated through multinomial logistic regression. Body height (OR: 1.05, 95% CI: 1.05–1.06), body mass index (OR: 0.98, 95% CI: 0.96–1.00), and ocular alignment (horizontal heterophoria vs. orthophoria, OR: 2.37, 95% CI: 2.08–2.70; tropia vs. orthophoria, OR: 1.94, 95% CI: 1.50–2.52) were significant factors for myopia in the elementary school group after adjustment for confounding factors. (Table 3) Only body height (OR: 1.10, 95% CI: 1.07–1.23) and ocular alignment (horizontal heterophoria vs. orthophoria, OR: 9.79, 95% CI: 5.06–19.0; tropia vs. orthophoria, OR: 16.8, 95% CI: 7.57–37.3) were significant factors for high myopia in the elementary school group. In the junior high school group, body height and ocular alignment were significant factors for myopia, and only ocular alignment (horizontal heterophoria vs. orthophoria, OR: 6.36, 95% CI: 2.24–18.1; tropia vs. orthophoria, OR: 15.3, 95% CI: 4.06–57.7) was significant for high myopia.

**Table 3:** Multinomial logistic regression of associated factors of myopia and high myopia in elementary and junior high school students (*n* = 5417)

	Myopia (*n* = 1718) vs. No Myopia (*n* = 2972)		High Myopia (*n* = 73) vs. No Myopia (*n* = 2972)	
	OR	95%CI	OR	95%CI
**Elementary school (aged 7–12) (*n*** **= 4763)**				
Body Height (cm)	1.05	1.05–1.06	1.10	1.07–1.12
BMI (Kg/m^2^)	0.98	0.96–1.00	0.98	0.93–1.04
Intraocular pressure (IOP),OD (mmHg)	1.02	1.00–1.03	1.04	0.99–1.09
Horizontal heterophoria vs. Orthophoria	2.37	2.08–2.70	9.79	5.06–19.0
Tropia vs. Orthophoria	1.94	1.50–2.52	16.8	7.57–37.3
	Myopia (*n* = 340) vs. No Myopia (*n* = 289)		High Myopia (*n* = 25) vs. No Myopia (*n* = 289)	
	OR	95%CI	OR	95%CI
**Junior high school (aged 13–15) (*n*** **= 654)**				
Body Height (cm)	1.02	1.00–1.03	1.03	0.98–1.08
BMI (Kg/m^2^)	1.03	0.99–1.07	1.07	0.98–1.16
Horizontal heterophoria vs. Orthophoria	2.20	1.56–3.10	6.36	2.24–18.1
Tropia vs. Orthophoria	1.73	0.84–3.57	15.3	4.06–57.7

## Discussion

### Prevalence and associated factors of myopia

Despite the implementation of the myopia prevention programme, the prevalence of myopia remained high in the rural prefecture of Taiwan. Horizontal heterophoria was an associated factor for myopia in children in both elementary and junior high school groups, with ORs of 2.37 and 2.20, respectively. It was also associated with high myopia, with ORs of 9.79 and 6.36 for children in elementary and junior high school groups, respectively. Rose et al. and Dirani et al. suggested that outdoor activity is an independent factor negatively associated with myopia [10, 11]. Thus, a strategy based on these findings can be developed to prevent myopia and high myopia [12]. Although the Ministry of Education in Taiwan launched a project using these very strategies in 2009, our study demonstrated that the prevalence of myopia remained high. This may be possible because of children being increasingly exposed to computers, communication and electronic consumer products, and after-school tutoring classes in Taiwan. Meanwhile, controversy exists whether near work is associated with myopia [13–15].

Our results demonstrated an increase in the prevalence of myopia with increasing age, which is compatible with the results in the literature [16]. The literature also shows that the annual incidence of myopia increased with age from 19.1% at aged 7 years to 30.2% at aged 11 years [16]. The increase in the prevalence with age would not be solely accumulation of myopic children, but also the increasing new myopia with age [16]. Therefore, more effort is needed in preventing new myopia as well as delaying the onset of myopia.

The association between myopia prevalence and horizontal heterophoria was further investigated in our study. Heterophoria was previously suggested to be associated with myopia in pre-school and elementary school children [17–20]. We demonstrated a close link between horizontal heterophoria and myopia. Children with heterophoria may progress to myopia though convergence accommodation [21–24]. Reduction of concomitant accommodation has been found to delay the progression of moderate myopia [25]. However, because we were unable to determine whether heterophoria is a risk factor for myopia or vice versa, further study is needed to clarify the link between myopia and heterophoria [18].

### Methodological considerations

This study has several limitations. First, we used noncycloplegic refraction because this was a population-based screening study, and cycloplegia was not well accepted by children and their parents. Choong et al. reported that autorefractors had a tendency towards minus overcorrection under noncycloplegic conditions [26]. Therefore, we used − 1.0 D to define myopia. The noncycloplegic autorefraction test was more convenient for vision-screening than the best-corrected visual acuity test and cycloplegic refraction test [9]. Second, we only evaluated a rural region in Taiwan. The prevalence of myopia is usually higher in urban areas than in rural areas. However, our results showed a high prevalence of myopia in the rural region, which warrants further evaluation. The study still retained sufficient statistical power to evaluate the presence of various risk factors for myopia given the rather large sample size. Finally, because our measurements were performed at a single point in time, they may not reflect long-term exposure to important demographic or biochemical factors.

## Conclusion

The prevalence of myopia was high at 40.1 and 55.8% among elementary school and junior high school groups, respectively, in the rural area of Chia-Yi. Heterophoria was associated with myopia and high myopia.

## Supplementary information

**Additional file 1.** Questionnaire. This document represented the questionnaire utilized in the present study.

## Data Availability

The datasets analysed during the current study are not publicly available for confidentiality reasons; however, the corresponding author will provide them on reasonable request.

## Abbreviations

SE: Spherical equivalent
OR: Odds ratio
