# Supplementary material for: Prevalence and associated factors of myopia among rural school students in Chia-Yi, Taiwan
Source: BMC Ophthalmol. 2020 Aug 5;20:320. doi: 10.1186/s12886-020-01590-y (PMC7405435; doi:10.1186/s12886-020-01590-y)
Supplement: Supplementary file 1 — Additional file 1. Questionnaire. This document represented the questionnaire utilized in the present study. [file 12886_2020_1590_MOESM1_ESM.docx]

**Questionnaire**

Name:

Age:

Class:

Gender:

Body weight (Measured)

Body height (Measured)

Duration of Sleep: □ >8 hours □ <8 hours

Eye related diseases or symptoms

- Headache
- History of physical disability
- Strabismus
- Amblyopia
